# Williams-Beuren syndrome associated with single kidney and nephrocalcinosis: a case report

**DOI:** 10.11604/pamj.2015.22.276.7929

**Published:** 2015-11-23

**Authors:** Kamel Abidi, Manel Jellouli, Rania Ben Rabeh, Yousra Hammi, Tahar Gargah

**Affiliations:** 1 Pediatric Nephrology Department, Charles Nicolle Hospital, Tunis, Tunisia

**Keywords:** Williams-Beuren syndrome, nephrocalcinosis, infant

## Abstract

Williams-Beuren syndrome is a rare neurodevelopmental disorder, characterized by congenital heart defects, abnormal facial features, mental retardation with specific cognitive and behavioral profile, growth hormone deficiency, renal and skeletal anomalies, inguinal hernia, infantile hypercalcaemia. We report a case with Williams-Beuren syndrome associated with a single kidney and nephrocalcinosis complicated by hypercalcaemia. A male infant, aged 20 months presented growth retardation associated with a psychomotor impairment, dysmorphic features and nephrocalcinosis. He had also hypercalciuria and hypercalcemia. Echocardiography was normal. DMSA renal scintigraphy showed a single functioning kidney. The FISH generated one ELN signal in 20 metaphases read and found the presence of ELN deletion, with compatible Williams-Beuren syndrome.

## Introduction

Williams-Beuren syndrome (WBS) was first identified in 1961 by Williams [1]. This syndrome is a rare neurodevelopmental disorder that occurs in 1 per 20000 live births. The occurrence is usually sporadic in most families. Approximately 90% of patients with Williams-Beuren syndrome have a deletion at chromosome 7q11.23, which can be detected by FISH (fluorescent in situ hybridization) analysis [2]. This micro deletion leads to the suppression of many genes, mainly the elastin gene. Williams-Beuren syndrome has often been associated with congenital heart defect, distinctive facial features, mental retardation with specific cognitive and behavioral profile, growth deficiency, renal and skeletal anomalies, inguinal hernia and infantile hypercalcaemia [2]. Several studies have focused on the types of renal and urinary tract anomalies in the Williams-Beuren syndrome. The frequency of these anomalies varies in the literature from 3% to 86% [3]. We report an original case of Williams-Beuren syndrome associated with a single kidney and nephrocalcinosis complicated hypercalcaemia.

## Patient and observation

**Figure 1:**
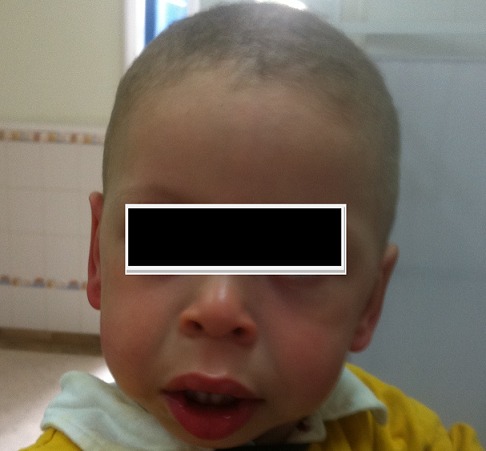
Williams-Beuren syndrome, dysmorphic features

This paper present a male infant, aged 20 months, born of consanguineous marriage, without special perinatal history, that was referred to the Pediatric Nephrology department because of growth retardation associated with a psychomotor impairment, dysmorphic features and nephrocalcinosis. Birth weight was 3200 g and the height was 48 cm. He had psychomotor retardation: smile-response acquired five months, holding the head at 10 months, sitting at 15 months, standing at 20 months and walking not yet acquired. At the age of 3 months, he had a bilateral inguinal hernia surgery. From the age of four months he had hypotonia, chronic vomiting associated with constipation and poor weight gain. The upper gastrointestinal endoscopy was normal. An abdominal ultrasound showed a rudimentary right kidney and a normal-sized left kidney with nephrocalcinosis, without dilatation of the excretory cavities. Admission physical examination found a very characteristic face: flat nasal bridge, everted lower lip, full cheeks, epicanthus, retrognathia, low-set ears and high arched palate. We did not find abnormal bones or spinal deformities (Figure 1). The weight and size were at three standard deviations below the mean and the head circumference was at one standard deviation above the mean. Cardiac auscultation was normal; blood pressure was suitable for age. He had hypercalcaemia (2.83 mmol/l) with normal creatinine level (24 µmol/l). The urine test showed hypercalciuria with a urinary calcium / urinary creatinine ratio: 1.627 (normal ratio <0.5). Echocardiography was normal. Ophthalmologic examination was normal. Thyroid tests were normal. DMSA renal scintigraphy showed a single left kidney in its normal place. The diagnosis of Williams-Beuren syndrome was defined and confirmed by genetics. Indeed, the fluorescence in situ hybridization test using MD Williams-Beuren Kreatech probe, specific for the ELN locus 7q11, was performed. The FISH generated one ELN signal in 20 metaphases read and found the presence of deletion of ELN locus, compatible with Williams-Beuren syndrome. The diagnosis of Williams-Beuren syndrome was made, and the patient is under observation, with a decline of eight months.

## Discussion

This paper is about an original case of Williams-Beuren syndrome associated with a single kidney and nephrocalcinosis complicated hypercalcaemia. This case illustrates several clinical features. First, in this case, cardiovascular abnormalities, which are present in 75% of patients, were absent. The typical cardiac defects are supravalvular aortic stenosis, supravalvular pulmonary stenosis and pulmonary artery stenosis. Supravalvular aortic stenosis occurs in approximately 70% of patients and is rare except in WBS. Intracardiac lesions, such as ventricular or atrial septal, defects are uncommon, whereas myxomatous degeneration of aortic or mitral-valve leaflets, or both, occurs in up to 20% of patients. Stenosis or coronary ostial occlusion can occur in absence of supravalvular aortic stenosis. Aortic collateral branches stenosis may also occur (coronary, cerebral or renal arteries). These vascular damages can lead to cerebral or cardiac ischemia [4]. At the histological level, WBS patients have stenosis of medium and large arteries led to thickening of vascular media, as a result of smooth-muscle overgrowth, collagen synthesis and intimal lesions, progressing to artery lumen occlusion. These patients have an imbalance in the ratio of matrix metalloproteinase / matrix metalloproteinase inhibitor (MMP-9/TIMP-1) with matrix degradation, which may facilitate smooth-muscle cells migration in the arterial wall and neointimal hyperplasia. Hypertension, occasionally starts in childhood, develops in approximately 50% of patients, as a result of stenosis of renal artery [5]. In addition, in the present case, the patient had hypercalcemia, hypercalciuria and nephrocalcinosis. Abnormalities in calcium homeostasis have been frequently described in WBS. These include hypocalcaemia which usually occurs during the first year of life, most cases resolve by four years of age, and hypercalciuria that may persist longer [6]. It has been reported that 5% to 50% of WBS patients, have one or more hypocalcaemia episodes. Hypocalcaemia is usually mild, though it can be moderate or severe, particularly during infancy. Hypercalcaemia can be asymptomatic or associated with nonspecific symptoms (hypotonia, constipation, abdominal pain …) that can even occur in WBS patients who have a normal calcium circulating level. Hypercalciuria is often accompanied by hypocalcaemia, but isolated hypercalciuria, especially it can occur after infancy. Nephrocalcinosis is relatively rare, found in less than 5% of WBS patients undergoing renal ultrasonography [6]. Various mechanisms have been suggested to explain the hypercalcaemia, but none have been confirmed. Hypercalcaemia may be due to intestinal hyper-absorption and / or decreased clearance of calcium. The increase of intestinal calcium absorption may be explained by increased 1,25-dihydroxy-vitamin D serum level. Another phenomenon occurred, decreased basal and Ca-stimulated calcitonin serum level [7]. More detailed investigations are required to discover the exact relation between WBS and Ca metabolism, especially during hypercalcemic phases.

## Conclusion

Performing a systematic laboratory and sonographic evaluation of the patients with Williams-Beuren syndrome is recommended.
